# Deep learning-based subtyping of gastric cancer histology predicts clinical outcome: a multi-institutional retrospective study

**DOI:** 10.1007/s10120-023-01398-x

**Published:** 2023-06-03

**Authors:** Gregory Patrick Veldhuizen, Christoph Röcken, Hans-Michael Behrens, Didem Cifci, Hannah Sophie Muti, Takaki Yoshikawa, Tomio Arai, Takashi Oshima, Patrick Tan, Matthias P. Ebert, Alexander T. Pearson, Julien Calderaro, Heike I. Grabsch, Jakob Nikolas Kather

**Affiliations:** 1 Else Kroener Fresenius Center for Digital Health, Technical University Dresden, Dresden, Germany; 2 Department of Medicine III, University Hospital RWTH Aachen, Aachen, Germany; 3 Department of Pathology, Christian-Albrechts University, Kiel, Germany; 4 Department of Visceral, Thoracic and Vascular Surgery, Technical University Dresden, University Hospital Carl Gustav Carus, Dresden, Germany; 5 Department of Gastric Surgery, National Cancer Center Hospital, Tokyo, Japan; 6 Department of Pathology, Tokyo Metropolitan Geriatric Hospital and Institute of Gerontology, Tokyo, Japan; 7 Department of Gastrointestinal Surgery, Kanagawa Cancer Center, Yokohama, Japan; 8 Duke-NUS Medical School, Singapore, Singapore; 9 Department of Medicine II, Medical Faculty Mannheim, Heidelberg University, Mannheim, Germany; 10 DKFZ-Hector Cancer Institute at the University Medical Center, Mannheim, Germany; 11 Clinical Cooperation Unit Healthy Metabolism, Center for Preventive Medicine and Digital Health, Medical Faculty Mannheim, Heidelberg University, Mannheim, Germany; 12 Mannheim Institute for Innate Immunoscience (MI3), Medical Faculty Mannheim, Heidelberg University, Mannheim, Germany; 13 Department of Medicine, Section of Hematology/Oncology, The University of Chicago, Chicago, IL USA; 14 Université Paris Est Créteil, INSERM, IMRB, Créteil, France; 15 Department of Pathology, Assistance Publique-Hôpitaux de Paris, Henri Mondor-Albert Chenevier University Hospital, Créteil, France; 16 Pathology & Data Analytics, Leeds Institute of Medical Research at St James’s, University of Leeds, Leeds, UK; 17 Department of Pathology, GROW School for Oncology and Reproduction, Maastricht University Medical Center+, Maastricht, The Netherlands; 18 Department of Medicine I, University Hospital Dresden, Dresden, Germany; 19 Medical Oncology, National Center for Tumor Diseases (NCT), University Hospital Heidelberg, Heidelberg, Germany

**Keywords:** Gastric cancer histology, Laurén classification, Deep learning classifier, Prognostic utility, Survival stratification, Hematoxylin, Eosin staining

## Abstract

**Introduction:**

The Laurén classification is widely used for Gastric Cancer (GC) histology subtyping. However, this classification is prone to interobserver variability and its prognostic value remains controversial. Deep Learning (DL)-based assessment of hematoxylin and eosin (H&E) stained slides is a potentially useful tool to provide an additional layer of clinically relevant information, but has not been systematically assessed in GC.

**Objective:**

We aimed to train, test and externally validate a deep learning-based classifier for GC histology subtyping using routine H&E stained tissue sections from gastric adenocarcinomas and to assess its potential prognostic utility.

**Methods:**

We trained a binary classifier on intestinal and diffuse type GC whole slide images for a subset of the TCGA cohort (*N* = 166) using attention-based multiple instance learning. The ground truth of 166 GC was obtained by two expert pathologists. We deployed the model on two external GC patient cohorts, one from Europe (*N* = 322) and one from Japan (*N* = 243). We assessed classification performance using the Area Under the Receiver Operating Characteristic Curve (AUROC) and prognostic value (overall, cancer specific and disease free survival) of the DL-based classifier with uni- and multivariate Cox proportional hazard models and Kaplan–Meier curves with log-rank test statistics.

**Results:**

Internal validation using the TCGA GC cohort using five-fold cross-validation achieved a mean AUROC of 0.93 ± 0.07. External validation showed that the DL-based classifier can better stratify GC patients' 5-year survival compared to pathologist-based Laurén classification for all survival endpoints, despite frequently divergent model-pathologist classifications. Univariate overall survival Hazard Ratios (HRs) of pathologist-based Laurén classification (diffuse type versus intestinal type) were 1.14 (95% Confidence Interval (CI) 0.66–1.44, *p*-value = 0.51) and 1.23 (95% CI 0.96–1.43, *p*-value = 0.09) in the Japanese and European cohorts, respectively. DL-based histology classification resulted in HR of 1.46 (95% CI 1.18–1.65, *p*-value < 0.005) and 1.41 (95% CI 1.20–1.57, *p*-value < 0.005), in the Japanese and European cohorts, respectively. In diffuse type GC (as defined by the pathologist), classifying patients using the DL diffuse and intestinal classifications provided a superior survival stratification, and demonstrated statistically significant survival stratification when combined with pathologist classification for both the Asian (overall survival log-rank test *p*-value < 0.005, HR 1.43 (95% CI 1.05–1.66, *p*-value = 0.03) and European cohorts (overall survival log-rank test *p*-value < 0.005, HR 1.56 (95% CI 1.16–1.76, *p*-value < 0.005)).

**Conclusion:**

Our study shows that gastric adenocarcinoma subtyping using pathologist’s Laurén classification as ground truth can be performed using current state of the art DL techniques. Patient survival stratification seems to be better by DL-based histology typing compared with expert pathologist histology typing. DL-based GC histology typing has potential as an aid in subtyping. Further investigations are warranted to fully understand the underlying biological mechanisms for the improved survival stratification despite apparent imperfect classification by the DL algorithm.

**Supplementary Information:**

The online version contains supplementary material available at 10.1007/s10120-023-01398-x.

## Introduction

The development and application of DL, Machine Learning (ML) and Artificial Intelligence-based methods and techniques has seen an exponential rise within the field of Computational Pathology over the past decade [1, 2]. In particular, computer vision-based DL techniques using routine H&E-stained tissue sections have opened potential avenues for clinically relevant DL-based assessment of H&E-stained specimens [3–6].

GC is among the most prevalent cancers worldwide [7], and the vast majority of GC are adenocarcinomas. Since its introduction in 1965, the histopathological classification system of Laurén (hereinafter referred to as Laurén) is one of the most widely used histological classifications for adenocarcinomas of the stomach in the West [8]. It identifies two main histological subtypes: intestinal and diffuse types. These subtypes are genetically distinct and associated with different clinical outcomes. In the advanced disease stage, diffuse type GC are typically considered to be more aggressive resulting in poorer patient prognosis compared with intestinal type GC [9]. Research has been performed investigating the utility of Laurén for patient management decisions and several clinical trials are currently ongoing randomizing patients to different treatment options based on Laurén [9–14].

GC is well known for its high degree of histological inter- and intratumoral heterogeneity which is most likely the reason for high rates of intra- and interobserver variability of all known GC histological classification systems including Laurén [15, 16]. The inter- and intratumoral heterogeneity of GC and resulting challenges in accurate and reproducible histological classification [17, 18] may render prediction of patient outcomes difficult. The 4th edition of the WHO classification defined for the first time a ‘mixed type’ GC as presence of a poorly cohesive component (diffuse type according to Laurén) in combination with another histological subtype irrespective of relative amounts of each component [19]. This may lead to a further increase in interobserver variation of GC histology classification. Furthermore, the variation in the extent of tumor tissue sampling may lead to potentially ‘misclassified’ cases.

GC incidence is highest in Asian countries. In addition, GC patients from these regions often have higher rates of diffuse type GC and as such many Asian medical societies have developed their own histological classification systems which is more similar to the WHO classification system than to Laurén [20]. That said, conversion tables between classifications have been published [21, 22].

To the best of our knowledge, while intestinal and diffuse type GC has been attempted to be predicted genetically [11], DL-based classification of GC into diffuse and intestinal type has not previously been attempted systematically using large cohorts with clinical follow-up data. We have previously used DL to predict diffuse and intestinal type GC, but these results, while encouraging, were limited to a technical benchmark study [23].

We hypothesized that DL has the potential to reduce the above-mentioned inter- and intra-observer variation in classifying histological subtypes in GC and thus may improve accuracy and reproducibility of Laurén through its usage as a classification aid for pathologists.

The primary objective of the present investigation was to (1) establish a DL-based model to classify GC as diffuse or intestinal type and to (2) test the model performance on digital H&E-stained tissue sections from GC resection samples from European and Asian patients without outlining tumor regions. The secondary objective was to determine the hypothetical utility of such a model when used alongside pathologist classifications.

## Methods

### Ethics statement and patient cohorts

This study was performed in accordance with the Declaration of Helsinki. This study is a retrospective analysis of digital images of anonymized archival tissue samples of multiple cohorts of GC patients. The overall analysis was approved by the Ethics board at University Hospital Carl Gustav Carus, Dresden, Germany. The collection of patient samples was approved by the Ethics board at each institution as described below. The KCCH cohort was obtained from the Kanagawa Cancer Center Hospital in Yokohama, Japan. The KIEL cohort was obtained from the Department of Pathology, University Hospital Schleswig–Holstein, Kiel, Germany and the analysis was approved by the local ethical review board (D 453/10) of University Hospital Schleswig–Holstein [24]. The clinicopathological characteristics, inclusion and exclusion criteria of the KIEL and KCCH cohorts have been previously described [24, 25]. All patients of both cohorts were treatment-naive at time of surgical resection; i.e. patients did not receive neoadjuvant chemotherapy. The remaining half of the KCCH cohort received adjuvant chemotherapy. General population characteristics of the cohorts are reported in Suppl. Table 1. This study adheres to the TRIPOD guidelines (Suppl. Table 2, Suppl. Figure 1) [26–28].

### Experimental design and statistics

**Fig. 1:**
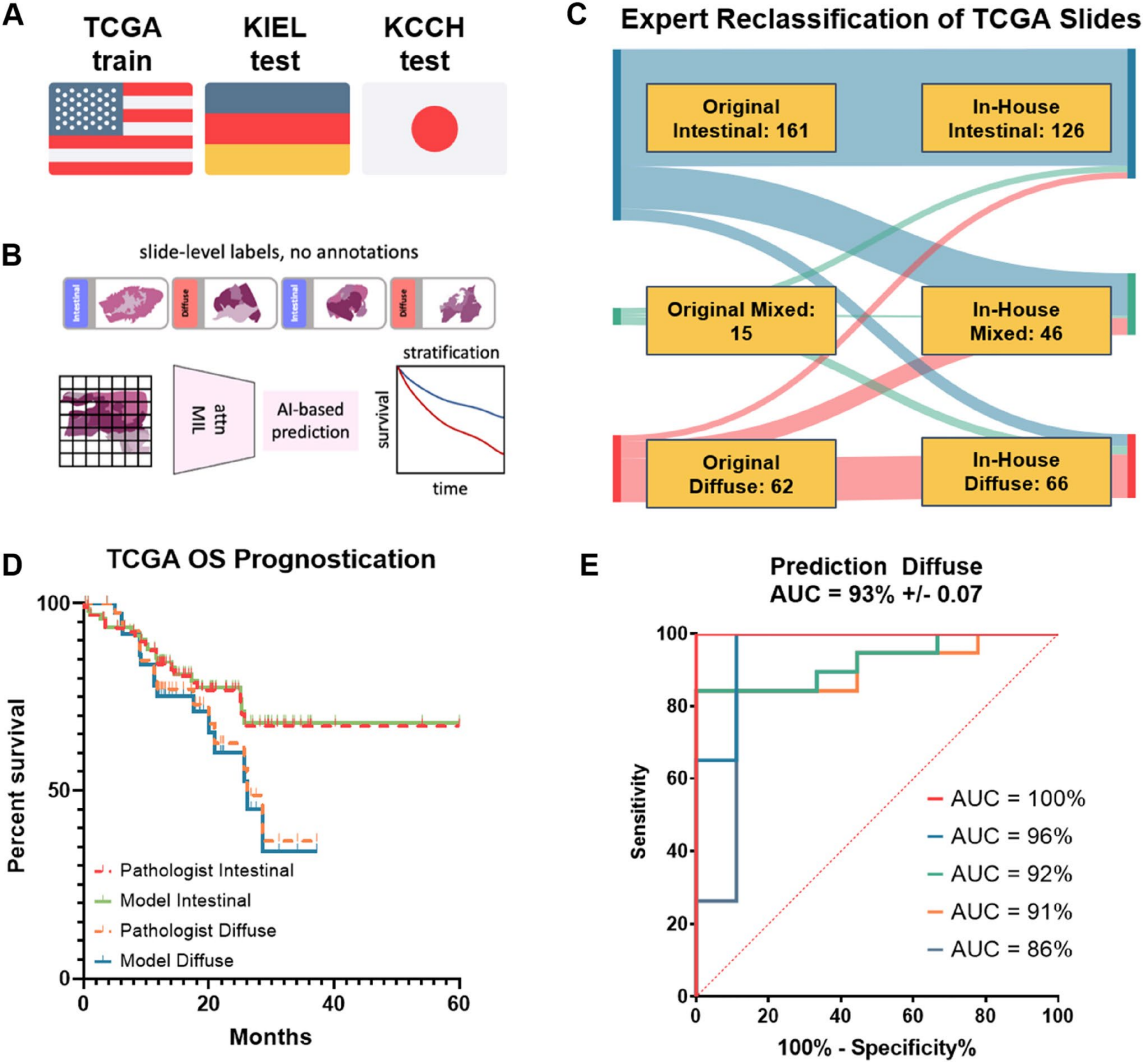
Outline of the study and development of the classifier. **A** The cohorts used for the present studies and their regional origin. **B** Overview of study methodology. **C** Sankey graph of how TCGA classifications changed when independently evaluated by our in-house expert pathologist (HIG). **D** KMC for OS comparing pathologist-model unanimity and discordance for intestinal and diffuse type using concatenated validation results for the TCGA cross-validation folds. **E** ROC curve for the five-fold cross validation on the TCGA cohort using only WSIs in which both TCGA and our pathologist were in unanimity with when performing Laurén

We trained all neural networks on the TCGA-GC (“TCGA-STAD'' in the original TCGA nomenclature) dataset via stratified five-fold cross-validation at patient-level (“within-cohort experimentation” for Laurén). Expert pathologists from Japan (TA) and the West (HIG) reclassified all available TCGA-GC H&E stained tissue sections (usually one per case) according to the Laurén (Suppl. Table 3). Only samples in which the original classification provided in the TCGA database and the revised classification were identical were used for training, validation and testing. The intention of the previous procedure was the reduction of interobserver variability in classification and thereby improving the quality of the ground truth. On the basis of acceptable performance defined as a mean AUROC of greater than 85% of the aforementioned folds with a lower standard deviation (SD) above 80%, we proceeded to train a model using the TCGA-GC dataset (N = 166). The model was externally validated on KCCH and KIEL separately (Fig. 1A). We estimated survival probabilities using Kaplan–Meier curves (KMCs) for both cohorts. The KMCs included those for: model predicted classifications, pathologist classifications, model and pathologist agreement for each subtype as well as model and pathologist disagreement for each subtype. We performed pairwise log-rank tests using Overall Survival (OS) and Cancer-Specific Survival (CSS) for KIEL, and OS, CSS and Disease-Free Survival (DFS) for KCCH using the aforementioned stratifiers. A *p* value of < 0.05 was considered statistically significant. No correction for multiple testing was applied.

### Image preprocessing

All images from H&E-stained tissue sections obtained from resection specimens used in our analysis were preprocessed according to the “Aachen protocol for deep learning histopathology” [29]. Whole Slide Images (WSIs) were obtained using Hamamatsu C13210 and Leica Aperio digital slide scanners for the KIEL and KCCH cohorts, respectively. WSIs were tessellated into image tiles of 256 µm edge length, processed at 224 by 224 pixel edge length (effective resolution of 1.14 μm per pixel), normalized according to ImageNet’s image statistics and augmented by rotation up to 360° and vertical flipping. Tiles not containing tissue and blurry tiles were automatically removed using the edge quantity as described in previous studies [30]. Tiles in the training set were color-normalized with the Macenko method [4, 31]. We applied a  DL technique known as attention Multiple Instance Learning (attMIL) [32], as described previously [33, 34], to train our model. AttMIL addresses a weakly supervised classification problem in which the objective is to predict a slide label from a collection of individual tiles. This mechanism assigns a weight to each tile, reflecting its importance for the classification task. The final case-based score is obtained by summing the product of vectors representing tiles generated by the embedding layer of the neural network and their corresponding attention weights and passing the resulting vector through a simple classifier. The attention mechanism allows our model to focus on the most informative regions within the whole slide image while considering the contribution of other tiles as well. We trained and tested a model on top of a frozen feature extractor trained with self-supervised learning. Wang et al. previously trained a ResNet-50 on 3200 WSIs from TCGA via the RetCCL self-supervised learning algorithm [35]. We used this pre-trained architecture to extract 2048 features per tile (Fig. 1B).

### Visualization and explainability

Visualization of morphological features relevant to the decision-making processes of DL models was important for: 1) identifying unique phenotypic patterns for different biomarkers and 2) better comprehension of how a model’s output was derived from its input data. For visualization, we plotted highly scoring tiles (top tiles) and whole slide heatmaps. The top tiles were the highest scoring tiles from patients that were correctly classified with the strongest confidence, i.e., with the highest registered probability scores obtained from passing individual tiles through the attMIL model [36]. Finally, slide heatmaps displayed distributions of the tiles’ attention and prediction scores over a WSI.

## Results

### Internal cross-validation of TCGA GC Laurén

The original TCGA database had *N* = 238 labeled samples (intestinal *n* = 161, diffuse *n* = 62, mixed *n* = 15). Two expert gastrointestinal pathologists (TA, HIG) reclassified the available H&E stained tissue sections as intestinal type *n* = 129, diffuse type *n* = 63 using Laurén or mixed *n* = 46 using the WHO classification. Only GC in which there was concordance between expert pathologist and original TCGA database were used for model training, testing and validation (Fig. 1C) leaving 166 GC (*n* = 116 intestinal type, *n* = 48 diffuse type and *n* = 2 mixed type). By doing so, we ensured that all training cases had a consistent and reliable classification that was established in a fully blinded manner, while still reflecting a consensus among the experts. As there were only two GC classified as mixed type in both classifications, these GC were excluded from further analyses.

We performed five-fold cross-validation on the TCGA-GC dataset and achieved a mean AUROC of 93% with a lower SD of 86% (Fig. 1D–E). As the performance met our predefined conditions, we proceeded to train a binary classifier (intestinal type versus diffuse type) on the TCGA GC dataset and used this model for all further external validation.

### DL-based histological GC classification and survival

**Fig. 2:**
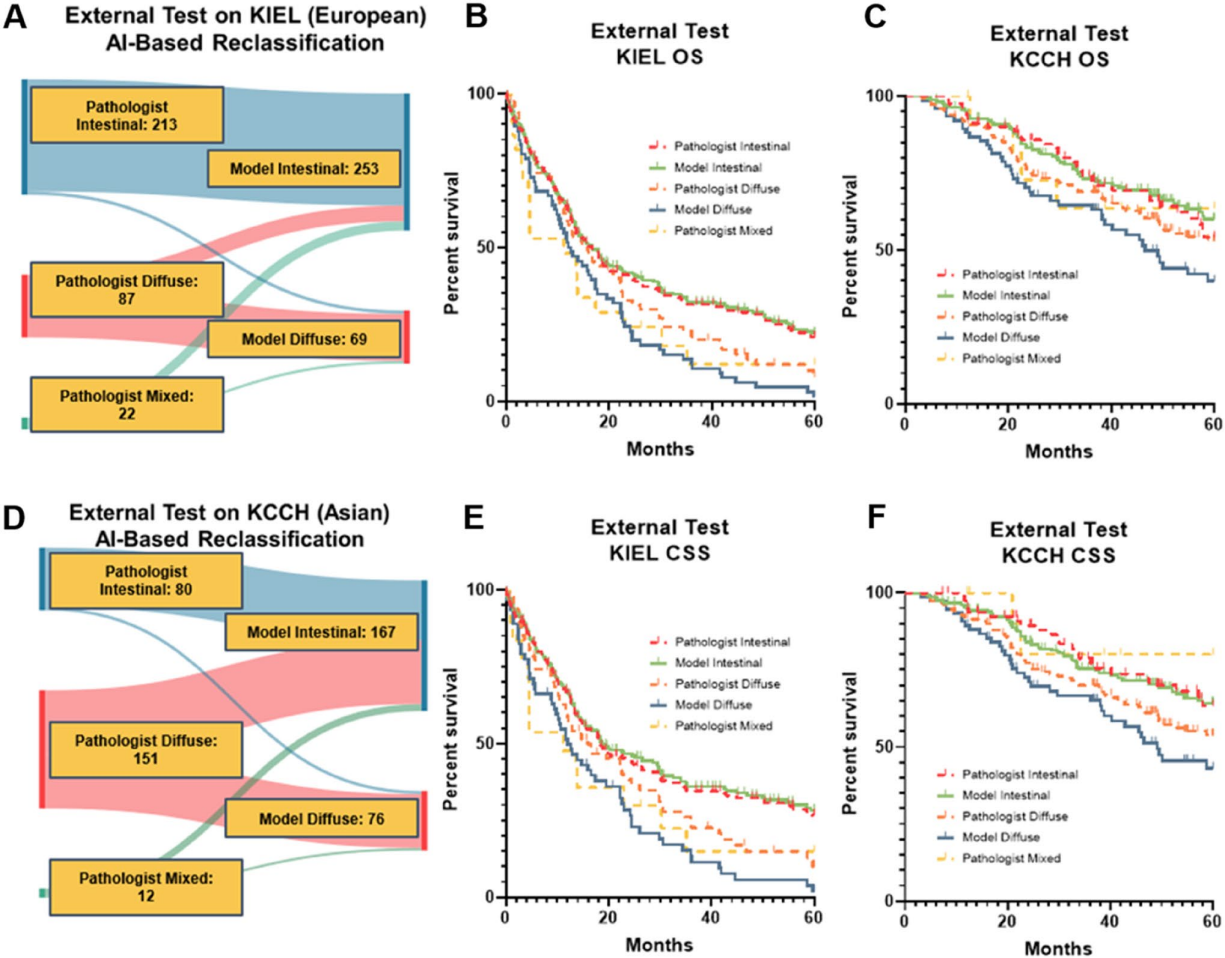
DL-based subtyping improves prognostication in external cohorts. **A** Sankey graph of how KIEL classifications changed when evaluated by our model **B** KMCs for 5-year OS comparing model classification with pathologist classification in the KIEL cohort. **C** KMCs for 5-year OS comparing model classification with pathologist classification in the KCCH cohort. **D** Sankey graph of how KCCH classifications changed when evaluated by our model. **E** KMCs for 5-year for CSS comparing model classification with pathologist classification in the KIEL cohort. **F** KMCs for CSS comparing model classification with pathologist classification in the KCCH cohort

Our single TCGA model was deployed on the KIEL and KCCH cohorts. *N* = 29 (33.3%) GC from the Kiel cohort and *N* = 82 (54.3%) GC from the KCCH cohort that were originally classified as diffuse type were reclassified as intestinal type by the DL model (Fig. 2A, D).

In the KCCH and the KIEL cohort, there was no significant difference in 5-year OS, CSS or DFS using the histological classification originally provided by the pathologist (intestinal type, diffuse type, mixed type) to stratify patients.

When using the DL-based histological classifier, there was a significantly poorer survival of the DL model-based diffuse type compared to the DL-based intestinal type in both cohorts (Fig. 2B, 2C, 2E, 2F, Suppl. Tables 4–5).

**Fig. 3:**
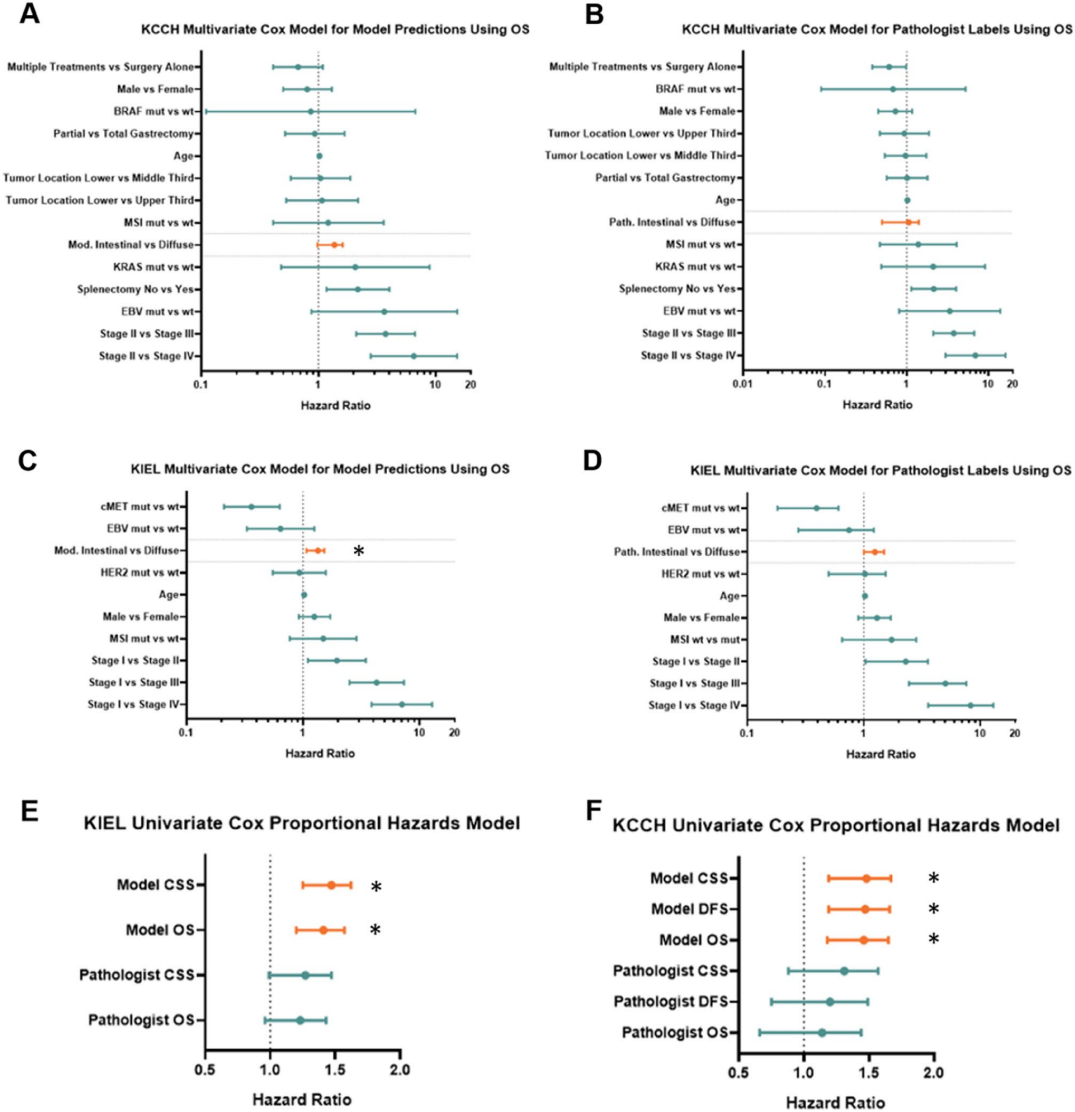
Forest plots for multivariate Cox proportional hazards models. **A** Forest plot for multivariate Cox proportional hazards model for 5-year overall survival in the KCCH Cohort using model predictions. **B** Forest plot for multivariate Cox proportional hazards model for 5-year overall survival in the KCCH cohort using pathologist classifications. **C** Forest plot for multivariate Cox proportional hazards model for 5-year overall survival in the KIEL cohort using model predictions. **D** Forest plot for multivariate Cox proportional hazards model for 5-year overall survival in the KIEL Cohort using pathologist classifications. **E** Forest plot for Cox proportional hazards model in the KIEL cohort. **F** Forest plot for Cox proportional hazards model in the KCCH cohort. Asterisks indicate *p*-values < 0.05

When performing univariate and multivariate (controlling for age, sex, UICC TNM stage, microsatellite instability (MSI), Epstein–Barr Virus (EBV)-, HER2- and cMET-status in the KIEL cohort, and in the KCCH cohort the aforementioned variables as well as tumor location, *KRAS*- and *BRAF*-mutation status, treatment type (surgery alone vs surgery followed by adjuvant chemotherapy), gastrectomy type and splenectomy status) Cox proportional hazards regression analyses, pathologist classification of diffuse and intestinal type GC was not related to survival (Suppl. Table 8, Suppl. Table 10). In contrast, univariate models stratifying patients by DL-based diffuse and intestinal type showed a significant relationship with all survival types in both cohorts. The DL-based classifier proved to be an independent prognostic marker in multivariate models in the KIEL cohort (Suppl. Table 11). While all multivariate models for KCCH outperformed their pathologist-labeled counterparts, none achieved a *p* value below 0.05 (*p* values of 0.06, 0.12 and 0.06 for OS, CSS and DFS, respectively) (Fig. 3A–D). Notably, even the lowest HR generated from DL-based classifier (multivariate CSS on the KCCH cohort) was higher than the best HR generated from pathologist-labeled data (univariate CSS on the KIEL cohort) (Fig. 3E–F).

### Interpretability of DL-based histological classification

**Fig. 4:**
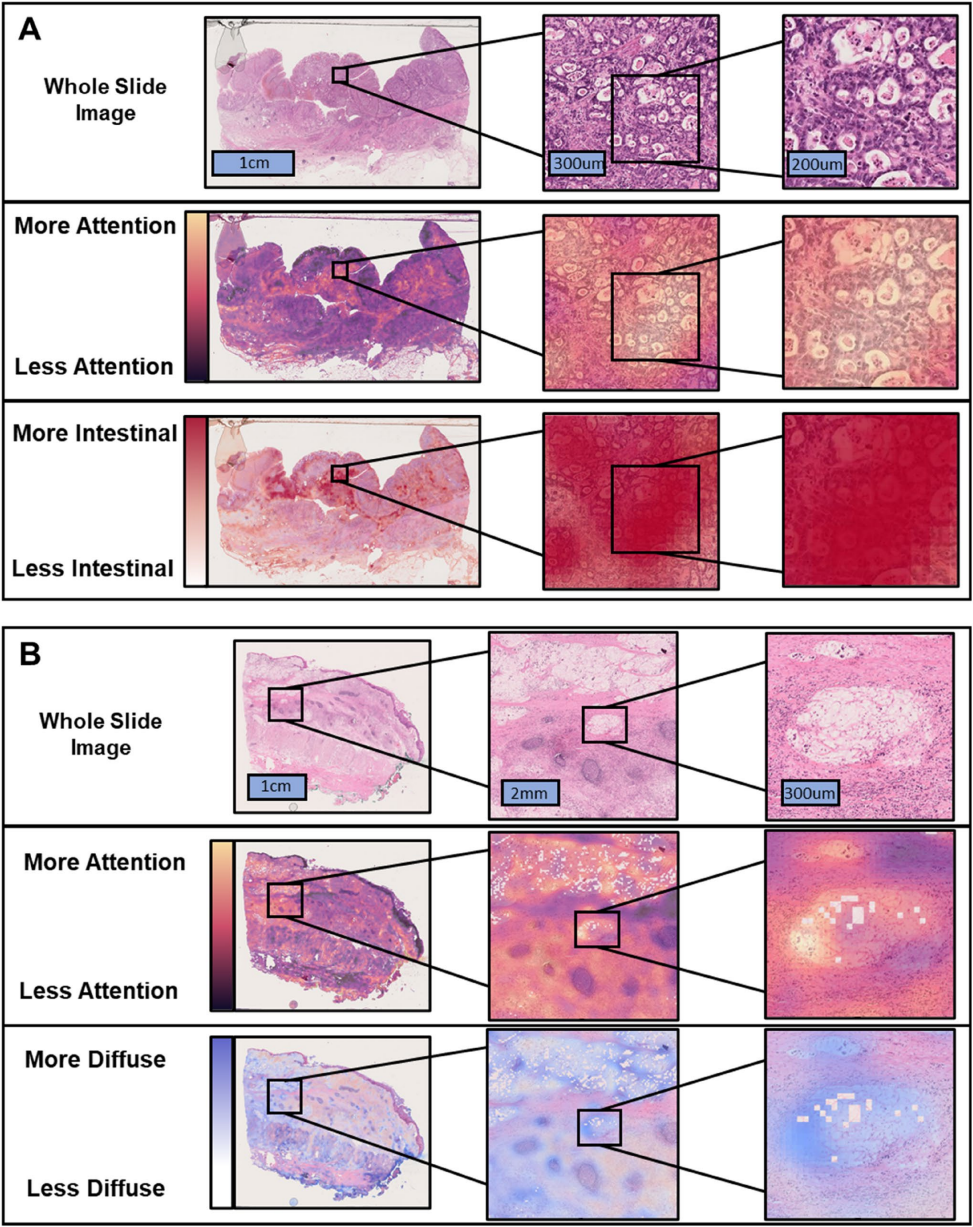
Interpretability of DL models. **A** Unaltered whole slide image along with the attention and classification maps for the same regions generated by our model for a slide labeled as intestinal the pathologist and diffuse by our model. Brighter regions in the attention map indicate greater importance by the model. Redder regions in the heat map indicate the model evaluating the region as increasingly intestinal type in nature. **B** Unaltered whole slide image along with the attention and classification maps for the same regions generated by our model for a slide labeled as intestinal the pathologist and diffuse by our model. Brighter regions in the attention map indicate greater importance by the model. Bluer regions in the heat map indicate the model evaluating the region as increasingly diffuse type in nature

We qualitatively assessed a selection of attention and heatmaps for WSI’s in which the DL-based classification disagreed with the pathologist’s classification, as well as WSI’s in which the pathologist’s classification was ‘mixed-type’. (Fig. 4A) It seemed that the disagreement between DL-based classification and pathologist’s classification might be more common in poorly differentiated cancer. In the cases classified as mixed type by the pathologist, the DL-based classifier mainly highlighted areas with intestinal type cancer. Due to the binary nature of the classifier, DL would categorize WSI’s containing intestinal and diffuse type features according to the majority of tiles falling in one of the categories. Furthermore, the presence of extracellular mucin in WSI’s led to inaccurate attention by the model negatively impacting the model’s accuracy (Fig. 4B). Additionally, artifacts related to cell death and autolysis appear to be misinterpreted as diffuse type GC by the DL model.

### Utility of model classifications in conjunction with pathologist classifications

**Fig. 5:**
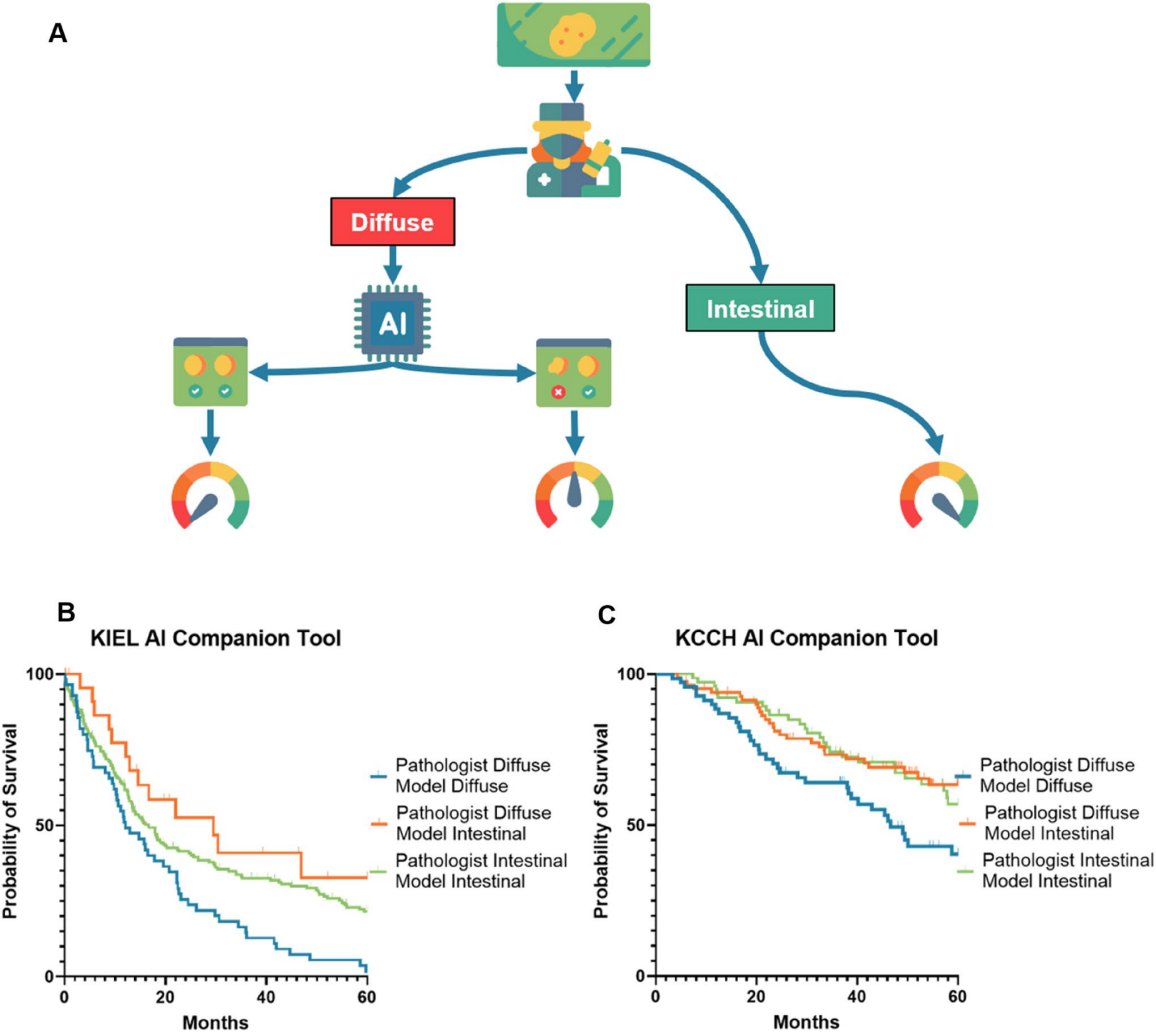
DL-based companion tool for improved clinical stratification. **A** Example workflow for how such a model can be used in assisting in patient stratification and prognostication. After an H&E slide is evaluated by a pathologist, if it has been labeled as diffuse type, it will then be handed over to the model for a second evaluation. In the event of pathologist-model unanimity, this patient will be stratified into the worst prognosis group. In the event of model-pathologist discordance, the patient will be stratified into the intermediate prognosis group. Finally if the patient is evaluated as intestinal type by the pathologist, this H&E slide will undergo no further assessment due to model-pathologist discordance being exceedingly rare and classified into the best prognosis group **B** Comparison of 5-year overall survival in the KIEL cohort when using the model as described in the first panel **C** Comparison of 5-year overall survival in the KCCH cohort when using the model as described in the first panel

As patient survival stratification seemed to be improved when using the DL-based classification in particular for DL-based diffuse type GC, we considered the model’s potential utility when combined with pathologist labeling so as to classify a GC as diffuse type with more certainty (Fig. 5A). We performed a subgroup analysis of patients classified as diffuse type by the pathologist (*n* = 87 and *n* = 151 for KIEL and KCCH respectively), and stratified patients into those where the model agreed with the pathologist’s classification and those where the model classified the cancer as intestinal type and investigated the relationship with survival of this new groupings. In both the KCCH and KIEL cohorts, we observed statistically significant survival stratification for all available survival types. We determined this through the log-rank tests as well as Cox proportional hazards regression. We observed a noticeable stratification in KMCs (Fig. 5B–C). Notably, the cases classified by the model as intestinal broadly follow the same survival trendline as the true intestinal population.

## Limitations

All specimens were obtained from resection specimens, however, tumor cell morphology changes from the tumor surface to the center and invasion front. Thus, it remains to be shown whether the DL-based classification can be applied to endoscopic tumor biopsies as treatment decisions for GC patients with resectable disease have to be made at the time of the diagnostic biopsy. Additionally, the highly heterogeneous nature of GC means that single tissue sections, as was used in the present study, may not be fully representative of the whole tumor. We also recognize that future work could benefit from investigating strategies for selecting the most representative sections of the tumor, taking into consideration the challenges posed by intratumoral heterogeneity in GC classification. Finally, we recognize the concern of overfitting in the TCGA cohort and acknowledge that some degree of overfitting is inevitable. We nonetheless believe that this study demonstrates the potential of DL-based histology typing as an aid in subtyping, serving as a strong foundation for further investigation.

## Discussion

Laurén is a commonly used method for histologic subtyping in GC. Nevertheless, this classification is subjective with high interobserver variation, and there remains a lack of consensus regarding its value as a prognostic tool. To the best of our knowledge, this is the first study to attempt Laurén prediction in GC using current state-of-the-art DL techniques in a systematic manner. Our results show that a model trained on TCGA GC WSIs using pathologist’s Laurén as ground truth achieves an excellent performance when internally cross-validated with an AUROC of 93%. Furthermore, we show that despite imperfect external validation performance, the DL-based histological subtype was able to stratify patients by survival whereas the pathologist-based Laurén (ground truth) did not. These findings seem to suggest that a DL-based histology subtype model trained on the pathologist’s Laurén classification and not trained on the survival data is able to predict patient survival. Up to 54% of GC classified as intestinal type by the DL model were originally classified as diffuse type by the pathologist. This relatively large number of reclassified cases using the DL-based classification could explain why the pathologist-based Laurén subtypes did not stratify patients by survival. This could reflect the known difficulties pathologists have to decide whether a poorly differentiated cancer should be classified as intestinal type or diffuse type. Further detailed quantitative analyses of the misclassified cases are needed to fully understand the prognostic relationship.

In our study, we observed a discrepancy between the high accuracy of the DL model in the TCGA dataset and its performance in the external datasets. However, it is important to emphasize that our primary objective was to assess the potential prognostic utility of the DL-based classifier for GC histology subtyping, rather than achieving perfect alignment with the Laurén classification. One has to realize that a DL-based model attempting to predict histological phenotype in GC will not fully reproduce the human decision-making process, e.g. the way pathologists establish the histological subtype according to the Lauren classification for a particular case. Pathologists may have a more nuanced view, evaluating the histological features on an intestinal-diffuse continuum to reach a final decision about the histological subtype. This strongly differs from the strictly dichotomous nature of the DL-based classifier based on the majority of patches classified as a certain subtype. Thus, because of the known heterogeneity of GC and the above explained majority call by the DL model, it is expected that during post-hoc evaluation of the DL-based classification by an expert pathologist, some of the DL model classification were found to be inaccurate. Nonetheless, survival stratification of patients appeared to be superior when using DL-based histological subtypes. This could indicate that the model is focused on a subset of histological features which happen to be prognostically relevant which are currently not recognized as such by the pathologist. It would be of clinical value to further scrutinize these histological slides, potentially using a different set of image analysis tools, to identify these prognostically relevant features so pathologists can learn to understand and recognize these themselves in the future. Alternatively, one could consider using a DL-based model to assist in the diagnostic process. The Laurén classification may not always be suitable for image analysis to predict the prognosis of patients. As an alternative, developing a ML-original classification could potentially yield better results. Consequently, we propose to investigate the development of such a classification system and compare its performance and similarity with conventional classification methods, including the Laurén classification. This would enable us to explore new avenues for predicting patient prognosis through image analysis, and could provide valuable insights for refining our current models.

In DL classification tasks within the field of computational histopathology, pathologist’s classification is generally treated as the ground truth when training the model [37]. Models are thus trained as best possible to imitate the pathologist’s classification. As a logical step from this, receiver operating characteristic curves and precision recall curves measuring the ability of the model to match pathologist classification are usually used as the primary form of model validation. However, in the case of certain classification tasks known to have high interobserver disagreement, one must consider carefully whether or not such methodologies are able to capture model performance accurately. Models are, as a general principle, only as good as the data they are trained, tested and validated on. There is an appreciable need for greater access and utilization of endpoints with greater objectivity such as survival, treatment response and other metrics with a lower propensity for imprecision and inaccuracies. These metrics are not without their own set of pitfalls, such a disagreement in treatment response or unclear cause of death due to a paucity of autopsies. However, these outcomes nonetheless serve to provide additional dimensions for interpreting the strengths and weaknesses of a given model. As such, when developing a model in which the ground truth is subjective and known for high levels of inter- and intra-observer disagreement, it may be worthwhile to incorporate such metrics to provide a more holistic perspective.

## Conclusion

Our study shows that gastric adenocarcinoma subtyping on the basis of the Laurén classification can be performed using current state of the art deep learning techniques. Our DL-based classifier was able to stratify patients by survival, whereas the pathologist-based histology subtype was unable to do so. This seems to be primarily driven by the model reclassifying many resections as intestinal type which were originally classified as diffuse type. Further validation in endoscopic biopsies and detailed investigation to identify the histological survival-relevant features recognized by the DL model but not the pathologist are warranted.

## Supplementary Information

Below is the link to the electronic supplementary material.

Supplementary file1 (DOCX 102 KB)

## Data Availability

All codes are open source and available at https://github.com/KatherLab/marugoto.

## Data availability

Some of the data that support the findings of this study are publicly available, and some are proprietary datasets provided under collaboration agreements. All data (including histological images) from the TCGA database are available at https://portal.gdc.cancer.gov/. All molecular data for patients in the TCGA cohorts are available at https://cbioportal.org/. All other data are under controlled access according to the local ethical guidelines and can only be requested directly from the respective study groups that independently manage data access for their study cohorts. Access to KIEL and KCCH was obtained via the Department of Pathology, Christian Albrechts University of Kiel, Kiel, Germany (https://www.medizin.uni-kiel.de/en/institutes-departments/institutes-of-clinical-theory/department-of-pathology) and the Kanagawa Cancer Center, Yokohama, Japan (https://kcch.kanagawa-pho.jp/en.html), respectively.
