# Supplementary material for: Deep learning-based subtyping of gastric cancer histology predicts clinical outcome: a multi-institutional retrospective study
Source: Gastric Cancer. 2023 Jun 3;26(5):708–20. doi: 10.1007/s10120-023-01398-x (PMC10361890; doi:10.1007/s10120-023-01398-x)
Supplement: Supplementary file 1 — Supplementary file1 (DOCX 102 KB) [file 10120_2023_1398_MOESM1_ESM.docx]

**Supplementary Tables**

|  | Age ± standard deviation | male:female | UICC Stages I:II:III:IV | Intestinal:  Diffuse:Mixed | MSI WT:MUT | EBV WT:MUT |
| --- | --- | --- | --- | --- | --- | --- |
| TCGA (N=235) | 61.1±10.9 | 141:82 | 32:83:89:20 | 162:62:15 | 189:49 | Unknown |
| KCCH (N=251) | 63.0±11.1 | 176:75 | 0:96:141:14 | 83:156:12 | 225:21 | 234:12 |
| KIEL (N=362) | 67.7±11.1 | 225:137 | 67:78:152:64 | 234:112:26 | 334:22 | 346:16 |

**Supplementary Table 1: Patient Characteristics.** This describes the general characteristics of the three cohorts as they were used for all stages of our testing. The EBV status of participants in the TCGA cohort is unknown. Due to the trial structure, the KCCH cohort had no UICC Stage I participants. Patient characteristics are otherwise similar between all three cohorts.

| **Section/Topic** | **Item** | **Checklist Item** | **Status** |
| --- | --- | --- | --- |
| Title and Abstract | | | |
| Title | 1 | Identify the study as developing and/or validating a multivariable prediction model, the target population, and the outcome to be predicted. | Reported |
| Abstract | 2 | Provide a summary of objectives, study design, setting, participants, sample size, predictors, outcome, statistical analysis, results, and conclusions. | Reported |
| Methods | | | |
| Source of data | 4a | Describe the study design or source of data (e.g., randomized trial, cohort, or registry data), separately for the development and validation data sets, if applicable | Reported and cited full description |
|  | 4b | Specify the key study dates, including start of accrual; end of accrual; and, if applicable, end of follow-up. | Cited full description |
| Participants | 5a | Specify key elements of the study setting (e.g., primary care, secondary care, general population) including number and location of centers. | Reported and cited full description |
|  | 5b | Describe eligibility criteria for participants. | Reported and cited full description |
|  | 5c | Give details of treatments received, if relevant. | Cited full description |
| Outcome | 6a | Clearly define the outcome that is predicted by the prediction model, including how and when assessed. | Reported |
|  | 6b | Report any actions to blind assessment of the outcome to be predicted. | Not applicable |
| Predictors | 7a | Clearly define all predictors used in developing or validating the multivariable prediction model, including how and when they were measured. | Reported |
|  | 7b | Report any actions to blind assessment of predictors for the outcome and other predictors. | Not applicable |
| Sample size | 8 | Explain how the study size was arrived at. | Reported |
| Missing data | 9 | Describe how missing data were handled (e.g., complete-case analysis, single imputation, multiple imputation) with details of any imputation method. | Cited full description |
| Statistical analysis methods | 10a | Describe how predictors were handled in the analyses. | Reported |
|  | 10b | Specify type of model, all model-building procedures (including any predictor selection), and method for internal validation. | Reported |
|  | 10d | Specify all measures used to assess model performance and, if relevant, to  compare multiple models. | Reported |
| Risk groups | 11 | Provide details on how risk groups were created, if done. | Reported |
| Results | | | |
| Participants | 13a | Describe the flow of participants through the study, including the number of participants with and without the outcome and, if applicable, a summary of the follow-up time. A diagram may be helpful. | Reported |
|  | 13b | Describe the characteristics of the participants (basic demographics, clinical features, available predictors), including the number of participants with missing data for predictors and outcome. | Reported |
| Model development | 14a | Specify the number of participants and outcome events in each analysis. | Reported |
|  | 14b | If done, report the unadjusted association between each candidate predictor and outcome. | Not applicable |
| Model specifications | 15a | Present the full prediction model to allow predictions for individuals (i.e., all regression coefficients, and model intercept or baseline survival at a given time point). | Reported |
|  | 15b | Explain how to use the prediction model. | Reported |
| Model performance | 16 | Report performance measures (with CIs) for the prediction model. | Reported |
| Discussion | | | |
| Limitations | 18 | Discuss any limitations of the study (such as non-representative sample, few events per predictor, missing data). | Reported |
| Interpretation | 19b | Give an overall interpretation of the results, considering objectives, limitations, and results from similar studies, and other relevant evidence. | Reported |
| Implications | 20 | Discuss the potential clinical use of the model and implications for future research. | Reported |
| Other information | | | |
| Supplementary information | 21 | Provide information about the availability of supplementary resources, such as study protocol, Web calculator, and data sets. | Reported |
| Funding | 22 | Give the source of funding and the role of the funders for the present study. | Reported |

**Supplementary Table 2: Tripod Checklist.** To ensure that the reporting of our results were of sufficient accuracy, we followed the checklist provided in the Transparent Reporting of a Multivariable Prediction Model for Individual Prognosis or Diagnosis (TRIPOD) guidelines.

|  | In-House Pathologist | | | |
| --- | --- | --- | --- | --- |
| TCGA Labeling |  | Intestinal | Diffuse | Mixed |
|  | Intestinal | 116 | 10 | 35 |
|  | Diffuse | 5 | 48 | 9 |
|  | Mixed | 8 | 5 | 2 |

**Supplementary Table 3: Confusion Matrix of TCGA and In-House Pathologist Labeling.** WSI’s from the TCGA cohort come with Laurén classification included as part of the tabular data provided. We had an expert pathologist independently assess these WSI’s in a blinded fashion. This confusion matrix compares the overlap and disagreement with regard to the classification between the original TCGA classification and our expert pathologist’s classification.

| Log-Rank Test Pairings | | Overall Survival (log-rank test statistic) | Cancer Specific Survival (log-rank test statistic) | Disease Free Survival (log-rank test statistic) |
| --- | --- | --- | --- | --- |
| Pathologist Intestinal | Pathologist Diffuse | 0.52, p-value =0.47 | 2.55, p-value = 0.11 | 1.10, p-value = 0.29 |
| Model Intestinal | Model Diffuse | 8.44, p-value <0.005 | 9.69, p-value <0.005 | 8.53, p-value <0.005 |

**Supplementary Table 4. Log-Rank Pairs for KCCH Cohort.** Results of the log-rank statistic calculations using the 5-year survival data in the KCCH cohort comparing pathologist intestinal-type and diffuse-type labeling, as well as model intestinal-type and diffuse-type labeling.

| Log-Rank Test Pairings | | Overall Survival (log-rank test statistic) | Cancer Specific Survival (log-rank test statistic) |
| --- | --- | --- | --- |
| Pathologist Intestinal | Pathologist Diffuse | 2.79, p-value =0.09 | 3.47, p-value = 0.06 |
| Model Intestinal | Model Diffuse | 14.52, p-value <0.005 | 17.35, p-value <0.005 |

**SupplementaryTable 5. Log-Rank Pairs for KIEL Cohort.** Results of the log-rank statistic calculations using the 5-year survival data in the KIEL cohort comparing pathologist intestinal-type and diffuse-type labeling, as well as model intestinal-type and diffuse-type labeling.

|  | Hazard Ratio | Lower 95% CI | Upper 95% CI | p-value |
| --- | --- | --- | --- | --- |
| Multivariate Cancer Specific Survival | 1.31 | 0.74 | 1.62 | 0.23 |
| Multivariate Overall Survival | 1.39 | 0.9 | 1.66 | 0.1 |
| Univariate Overall Survival | 1.43 | 1.05 | 1.66 | 0.03 |
| Univariate Cancer Specific Survival | 1.47 | 1.11 | 1.68 | 0.02 |
| Multivariate Disease Free Survival | 1.47 | 1.04 | 1.7 | 0.04 |
| Univariate Disease Free Survival | 1.5 | 1.15 | 1.7 | 0.01 |

**Supplementary Table 6. Cox Regression for KCCH as a Diagnostic Aid, Comparing Diffuse Type Model-Pathologist Unanimity with Disagreement.** Results of the Cox Proportional-Hazards Model presented in tabular form for both univariate and multivariate analyses performed for the proposed diagnostic aid for all forms of survival data available in the KCCH cohort.

|  | Hazard Ratio | Lower 95% CI | Upper 95% CI | p-value |
| --- | --- | --- | --- | --- |
| Univariate Overall Survival | 1.28 | 0.52 | 1.65 | 0.37 |
| Multivariate Cancer Specific Survival | 1.36 | 0.6 | 1.7 | 0.27 |
| Univariate Overall Survival | 1.56 | 1.16 | 1.76 | <0.005 |
| Univariate Cancer Specific Survival | 1.61 | 1.21 | 1.8 | <0.005 |

**Supplementary Table 7. Cox Regression for KIEL as a Diagnostic Aid, Comparing Diffuse Type Model-Pathologist Unanimity with Disagreement.** Results of the Cox Proportional-Hazards Model presented in tabular form for both univariate and multivariate analyses performed for the proposed diagnostic aid for all forms of survival data available in the KIEL cohort.

| KIEL Multivariate for OS using Pathologist | Hazard Ratio | Lower 95% CI | Upper 95% CI | p-value |
| --- | --- | --- | --- | --- |
| cMET mut vs wt | 0.36 | 0.21 | 0.63 | <0.005 |
| EBV mut vs wt | 0.64 | 0.33 | 1.25 | 0.189 |
| Pathologist Intestinal vs Diffuse | 1.34 | 1.07 | 1.52 | <0.005 |
| HER2 mut vs wt | 093 | 0.55 | 157 | 0.786 |
| Age | 1.02 | 1.01 | 1.04 | 0.008 |
| Male vs Female | 1.25 | 092 | 1.71 | 0.158 |
| MSI mut vs wt | 1.49 | 0.77 | 2.87 | 0.235 |
| Stage I vs Stage II | 195 | 1.10 | 3.46 | 0.022 |
| Stage I vs Stage III | 4.28 | 2.50 | 7.33 | <0.005 |
| Stage I vs Stage IV | 7.04 | 3.87 | 12.79 | <0.005 |

**Supplementary Table 8. Multivariate Cox Regression for KIEL using Pathologist Laurén Classification.** Results of the multivariate Cox Proportional-Hazards Model presented in tabular form. This uses the pathologist’s labeling of diffuse and intestinal as opposed to the model’s.

| KIEL Multivariate for OS using Model | Hazard Ratio | Lower 95% CI | Upper 95% CI | p-value |
| --- | --- | --- | --- | --- |
| cMET mut vs wt | 0.36 | 0.21 | 0.63 | <0.005 |
| EBV mut vs wt | 0.64 | 0.33 | 1.25 | 0.2075 |
| Model Intestinal vs Diffuse | 1.34 | 1.07 | 1.52 | 0.0198 |
| HER2 mut vs wt | 0.93 | 0.55 | 1.57 | 0.7875 |
| Age | 1.02 | 1.01 | 1.04 | 0.0080 |
| Male vs Female | 1.25 | 0.92 | 1.71 | 0.1477 |
| MSI mut vs wt | 1.49 | 0.77 | 2.87 | 0.2198 |
| Stage I vs Stage II | 1.95 | 1.10 | 3.46 | 0.0128 |
| Stage I vs Stage III | 4.28 | 2.50 | 7.33 | <0.005 |
| Stage I vs Stage IV | 7.04 | 3.87 | 12.79 | <0.005 |

**Supplementary Table 9. Multivariate Cox Regression for KIEL using Model Laurén Classification.** Results of the multivariate Cox Proportional-Hazards Model presented in tabular form. This uses the model’s labeling of diffuse and intestinal as opposed to the pathologist’s.

| KCCH Multivariate for OS using Pathologist | Hazard Ratio | Lower 95% CI | Upper 95% CI | p-value |
| --- | --- | --- | --- | --- |
| Multiple Treatments vs Surgery Alone | 0.61 | 0.38 | 0.99 | 0.043 |
| Male vs Female | 0.68 | 0.09 | 5.26 | 0.710 |
| BRAF mut vs wt | 0.73 | 0.45 | 1.17 | 0.197 |
| Partial vs Total Gastrectomy | 0.94 | 0.47 | 1.88 | 0.861 |
| Age | 0.97 | 0.54 | 1.74 | 0.919 |
| Tumor Location Lower vs Middle Third | 1.01 | 0.57 | 1.8 | 0.973 |
| Tumor Location Lower vs Upper Third | 1.02 | 1 | 1.04 | 0.048 |
| MSI mut vs wt | 1.06 | 0.5 | 1.41 | 0.826 |
| Pathologist Intestinal vs Diffuse | 1.39 | 0.47 | 4.09 | 0.551 |
| KRAS mut vs wt | 2.12 | 0.49 | 9.12 | 0.314 |
| Splenectomy No vs Yes | 2.15 | 1.14 | 4.03 | 0.017 |
| EBV mut vs wt | 3.37 | 0.81 | 14 | 0.095 |
| Stage II vs Stage III | 3.77 | 2.12 | 6.72 | <0.005 |
| Stage II vs Stage IV | 6.96 | 2.99 | 16.21 | <0.005 |

**Supplementary Table 10. Multivariate Cox Regression for KCCH using Pathologist Laurén Classification**. Results of the multivariate Cox Proportional-Hazards Model presented in tabular form. This uses the pathologist’s labeling of diffuse and intestinal as opposed to the model’s.

| KCCH Multivariate for OS using Model | Hazard Ratio | Lower 95% CI | Upper 95% CI | p-value |
| --- | --- | --- | --- | --- |
| Multiple Treatments vs Surgery Alone | 0,67 | 0,41 | 1,09 | 0,108 |
| Male vs Female | 0.8 | 0.5 | 1.3 | 0.360 |
| BRAF mut vs wt | 0.86 | 0.11 | 6.7 | 0.886 |
| Partial vs Total Gastrectomy | 0.93 | 0.52 | 1.67 | 0.807 |
| Age | 1.02 | 1 | 1.04 | 0.048 |
| Tumor Location Lower vs Middle Third | 1.04 | 0.58 | 1.87 | 0.896 |
| Tumor Location Lower vs Upper Third | 1.07 | 0.53 | 2.18 | 0.851 |
| MSI mut vs wt | 1.21 | 0.41 | 3.6 | 0.731 |
| Model Intestinal vs Diffuse | 1.37 | 0.98 | 1.61 | 0.013 |
| KRAS mut vs wt | 2.06 | 0.48 | 8.86 | 0.331 |
| Splenectomy No vs Yes | 2.17 | 1.17 | 4.03 | 0.014 |
| EBV mut vs wt | 3.64 | 0.87 | 15.26 | 0.077 |
| Stage II vs Stage III | 3.74 | 2.1 | 6.66 | <0.005 |
| Stage II vs Stage IV | 6.51 | 2.79 | 15.22 | <0.005 |

**Supplementary Table 11. Multivariate Cox Regression for KCCH using Model Laurén Classification.** Results of the multivariate Cox Proportional-Hazards Model presented in tabular form. This uses the model’s labeling of diffuse and intestinal as opposed to the pathologist’s.

|  | Log-Rank Test Pairings | | Overall Survival (log-rank test statistic) | Cancer Specific Survival (log-rank test statistic) | Disease Free Survival (log-rank test statistic) |
| --- | --- | --- | --- | --- | --- |
| KIEL, Pathologist Labeled Diffuse Type | Model Diffuse Label | Model Intestinal Label | 8.87, p-value<0.005 | 9.19, p-value<0.005 | Not Applicable. |
| KCCH, Pathologist Labeled Diffuse Type | Model Diffuse Label | Model Intestinal Label | 6.34, p-value=0.01 | 5.15, p-value=0.02 | 7.15, p-value=0.01 |

**Supplementary Table 12. Log-Rank Pairs for Pathologist Diffuse Type Subgroups for both KCCH and KIEL.** Results of the log-rank statistic calculations using the 5-year survival data for both the KIEL and KCCH cohorts looking exclusively at WSI’s that were labeled as diffuse-type by the pathologist. Within this subgroup we compared those that the model classified as diffuse-type as well and those that the model disagreed with the pathologist and labeled as intestinal-type instead.


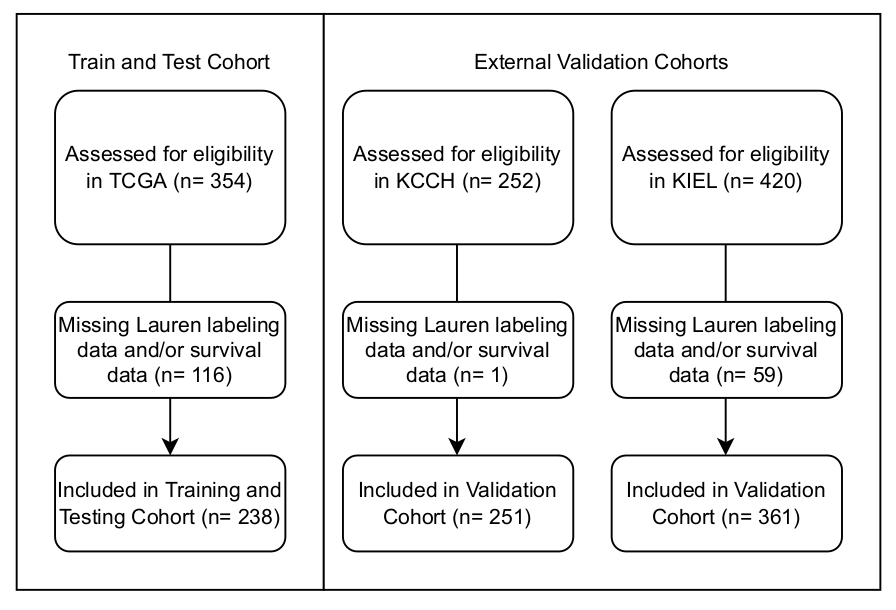


**Supplementary Figure 1. Modified CONSORT Flow Diagram.** A modified Consolidated Standards of Reporting Trials (CONSORT) flow diagram to describe the reasons for participant exclusion in analysis as well as the number of patients before and after exclusion.
